# Shedding and exclusion from childcare in children with Shiga toxin-producing *Escherichia coli*, 2018–2022

**DOI:** 10.1017/S095026882400027X

**Published:** 2024-02-26

**Authors:** Amoolya Vusirikala, Sam Rowell, Girija Dabke, Georgina Fox, Jade Bell, Rohini Manuel, Claire Jenkins, Nicola Love, Noel McCarthy, Dana Sumilo, Sooria Balasegaram

**Affiliations:** 1 Health Protection Operations, UK Health Security Agency, London, UK; 2 UK Field Epidemiology Training Programme, UK Health Security Agency, London, UK; 3 Clinical and Public Health Group, UK Health Security Agency, London, UK; 4 National Institute for Health and Care Research (NIHR) Health Protection Research Unit (HPRU) in Gastrointestinal Infection at University of Liverpool, Liverpool, UK; 5 School of Medicine, Trinity College Dublin, Dublin, Ireland; 6 Warwick Medical School, School of Medicine, Warwick, UK

**Keywords:** Shiga toxin-producing *Escherichia coli*, epidemiology, childcare setting, carriage, transmission

## Abstract

Excluding children with Shiga toxin-producing *Escherichia coli* (STEC) from childcare until microbiologically clear of the pathogen, disrupts families, education, and earnings. Since PCR introduction, non-O157 STEC serotype detections in England have increased. We examined shedding duration by serotype and transmission risk, to guide exclusion advice. We investigated STEC cases aged <6 years, residing in England and attending childcare, with diarrhoea onset or sample date from 31 March 2018 to 30 March 2022. Duration of shedding was the interval between date of onset or date first positive specimen and earliest available negative specimen date. Transmission risk was estimated from proportions with secondary cases in settings attended by infectious cases. There were 367 cases (STEC O157 *n* = 243, 66.2%; STEC non-O157 *n* = 124, 33.8%). Median shedding duration was 32 days (IQR 20–44) with no significant difference between O157 and non-O157; 2% (*n* = 6) of cases shed for ≥100 days. Duration of shedding was reduced by 17% (95% CI 4–29) among cases reporting bloody diarrhoea. Sixteen settings underwent screening; four had secondary cases (close contacts’ secondary transmission rate = 13%). Shedding duration estimates were consistent with previous studies (median 31 days, IQR 17–41). Findings do not warrant guidance changes regarding exclusion and supervised return of prolonged shedders, despite serotype changes.

## Introduction

Shiga toxin-producing *Escherichia coli* (STEC) are defined by the presence of the Shiga toxin gene (*stx*). STEC are a significant public health concern due to the potential severity of disease and outbreaks. Symptoms can range from mild to severe bloody-stained diarrhoea and may include, abdominal pain, and nausea. Children are at increased risk of developing haemolytic uraemic syndrome (HUS), a systemic condition characterized by renal complications, that can be fatal [1]. STEC that have the potential to cause HUS typically have *stx* subtypes *stx2a* and/or *stx2d*, and in England most frequently include STEC serotypes O157:H7, O26:H11, and O145:H28.

STEC can be transmitted to humans through consumption of contaminated food or water, through direct contact with carrier animals or their faecal material, or through person-to-person transmission. Public health authorities frequently have to manage STEC cases, instigating interventions to prevent secondary person-to-person transmission.

A frequently used intervention strategy is the exclusion of children with STEC from childcare settings [2–5] until they are microbiologically clear of the infection. Children have increased susceptibility to acquiring and transmitting STEC infection, likely due to immature immune systems and underdeveloped hygiene practices and enhanced risk of developing HUS. In England, children infected with presumptive STEC O157 and non-O157 serogroups exhibiting virulence profiles associated with the potential to cause HUS are excluded until two consecutive negative clearance faecal specimens, taken at least 24 h apart, are obtained [6]. Exclusion can be lengthy when carriage in children is prolonged. Exclusion results in disruption to families in terms of potential loss of earnings, the child’s education, emotional and mental stress, and disengagement with the health system [7].

The greatest period of transmissibility is likely to be when cases are symptomatic, but the risk of secondary transmission is also influenced by the period of shedding of the pathogen. This is considered to be up to around 7 days in adults, but in children prolonged shedding may occur [6]. In the UK, evidence regarding the infectious dose, transmissibility, and duration of shedding of STEC by human cases has been derived principally from cases of STEC O157. A study of children attending childcare settings in England using 2010–2011 data estimated the median duration of shedding was 31 days, and 24% continued to shed for ≥6 weeks. Secondary cases occurred in 7% (6/83) of settings attended by infectious cases [8]. Ninety-nine per cent of cases in this study were STEC O157.

Since this study, the epidemiology of detected STEC has changed [9–11]. There has been an increase in notifications of non-O157 STEC, attributable in part in England to the improvements in diagnostic capabilities of local and regional laboratories following the implementation of commercial gastrointestinal PCR assays. It is estimated that for every one STEC O157 isolated, there are 4–7 other non-O157 STEC isolated, with various *stx* subtypes and virulence factors (*eae* gene), capable of causing illnesses from mild to severe, including HUS [6].

Few studies on STEC shedding duration and risk of secondary transmission in childcare settings by STEC serotype have been conducted, particularly outside of an outbreak context. Studies conducted in Sweden showed no differences in time to clearance between STEC *stx* types and presence or absence of *eae* [12, 13], but a recent study in Ireland identified a shorter length of time to achieve microbiological clearance among those infected with STEC O26 compared to STEC O157, although this was not a statistically significant finding [14].

With recent increased non-O157 notifications STEC in England, a reassessment of shedding duration, exclusion, and transmission is necessary. Updated carriage duration estimates in children and secondary transmission risk will inform implementation of control measures, guide the formulation of management plans with parents, and improve national guidelines.

## Methods

We carried out a population-based retrospective study of laboratory-confirmed STEC cases aged <6 years, attending a childcare facility. Cases with an onset date or sample date from 31 March 2018 to 30 March 2022 (inclusive) who resided in England at the time of diagnosis were eligible for inclusion in the study.

### Data sources

STEC is notifiable in England under the Health Protection (Notification) Regulations 2010. Data are recorded in two systems, and both were used to identify cases and extract required data items:

- The National Enhanced STEC surveillance system (NESSS) holds a standard set of clinical, epidemiological, and microbiological data for all STEC cases, captured via enhanced surveillance questionnaires (ESQs) and reconciled with laboratory reports linked to each case.
- A case management system records each case of STEC managed by health protection teams (HPTs). A survey developed in a web-based survey tool (Snap 11 Professional) was used to extract specific information from records including dates of clearance samples, date child was advised to return to setting, and whether the child was infectious at the setting.

### Definitions

- Childcare setting included nurseries, preschools, primary schools, and child minders/nannies.
- Microbiological clearance was defined as the absence of STEC in faecal specimens tested using culture-based methods at local laboratories, UKHSA regional laboratories, or GBRU.
- A case in this study was defined as a laboratory-confirmed case (STEC confirmed by culture, or PCR positive for stx genes, as reported by the GBRU) that normally attends a childcare setting.
- Primary case was defined as a symptomatic case with no history of close contact with a confirmed case in the 7 days prior to onset of illness.
- Co-primary case was a case with a date of onset within one incubation period (4 days) of the primary case, i.e., a case thought to have been exposed to the same infection source as the primary case.
- Secondary case was a case with a date of onset more than one incubation period (4 days) after the primary case, or where transmission was believed to be through exposure to a primary case.
- Child infectious at childcare setting was recorded if case records indicated that the child attended the facility symptomatic with diarrhoea, or definitely or probably shedding (identified from PCR test or culture).

### Data analysis

#### Shedding duration and factors associated with shedding duration

The duration of STEC carriage was estimated as the interval in days from date of onset of symptoms to the earliest available negative sample date. If the case was asymptomatic, carriage was calculated from the sample date of the first positive faecal specimen. If sample date missing, report date was used. Duration of shedding was defined in terms of median and interquartile range of days. Duration of shedding was also calculated excluding dates of second negative samples. Linear regression analysis was conducted to determine the association of factors such as age on log-transformed duration of shedding. Statistical tests including Wilcoxon and Kruskal–Wallis were used to compare the median duration of carriage by sex, organism serotype, *stx* pattern, clinical presentation, and any treatment (antibiotic and anti-diarrhoea) given.

#### Exclusion

The duration of required exclusion was calculated as the interval from date of onset to the date of the report of the second negative sample (if there was no second negative sample, the first sample date was used). The duration of actual exclusion was calculated as the interval from date of exclusion from the setting to date of phone call to carers of the case, indicating the case can return to the setting as recorded on the public health case management system. If date of exclusion was not available, date of onset was used as a proxy, as parents would have likely removed symptomatic children from childcare facilities. Where both duration of shedding and exclusion was known, the median duration of exclusion was compared to the median duration of shedding.

A sensitivity analysis was also conducted by including only individuals with a known date of exclusion to calculate the median duration of actual exclusion.

#### Transmission of STEC within childcare settings

The proportion of infectious children attending childcare settings in the study population and the duration of attendance while infectious was calculated.

Evidence of transmission occurring was recorded if at least one laboratory-confirmed secondary case was identified in a childcare setting attended by at least one infectious case. We describe each secondary transmission event (incident) by the type of setting, type of cases, and secondary transmission rate. The proportion of childcare settings where secondary transmission occurred was calculated by dividing the number of settings with two or more laboratory-confirmed cases by the total number of settings that had been attended by at least one infectious case. In settings where close contact screening took place, the secondary transmission rate among close contacts was calculated from the number of secondary cases in close contacts divided by the number of close contacts.

Data were processed and analysed using STATA version 17.

## Results

There were 1,033 confirmed cases of STEC in children aged <6 years old reported in England between 31 March 2018 and 30 March 2022 (Supplementary Figure 1). Of the 850/1,033 (82%) of cases with a known serotype, 367/850 (36%) had evidence of attending a childcare setting from the ESQ and were included in the study.

In the study population, the median age was three (IQR 2–4) years and 50.4% (*n* = 185) of the children were male. Of the 274 (74.6%) cases with information available on ethnicity, 218 (79.6%) were White, 25 (9.^1^%) were Asian or Asian British, 9 (3.3%) were Black or Black British, and 22 (8.^3^%) were mixed or other.

Symptoms reported included diarrhoea (95.5%, 315/330 cases where information known), and/or bloody diarrhoea (46.8%, 141/301 cases). There were 37/367 (10.1%) cases reported to have developed HUS, two of these cases died. There were 34 (9.^3^%) asymptomatic cases identified via contact screening. Of the 367 cases, 243 (66.2%) had STEC O157 and 124 (33.8%) had non-O157 (two most common – O26:H11, *n* = 74/124, 59.7%; O145:H28 *n* = 9/124, 2.5%). Of the 367 STEC isolated, 95% (348/367) had *eae* positive and 91% (333/366; 1 missing *stx* profile) had *stx2*, of which 96% (320/333) were also *eae* positive.

### Duration of STEC carriage in children aged 5 years or less, who attend childcare settings

**Figure 1.**
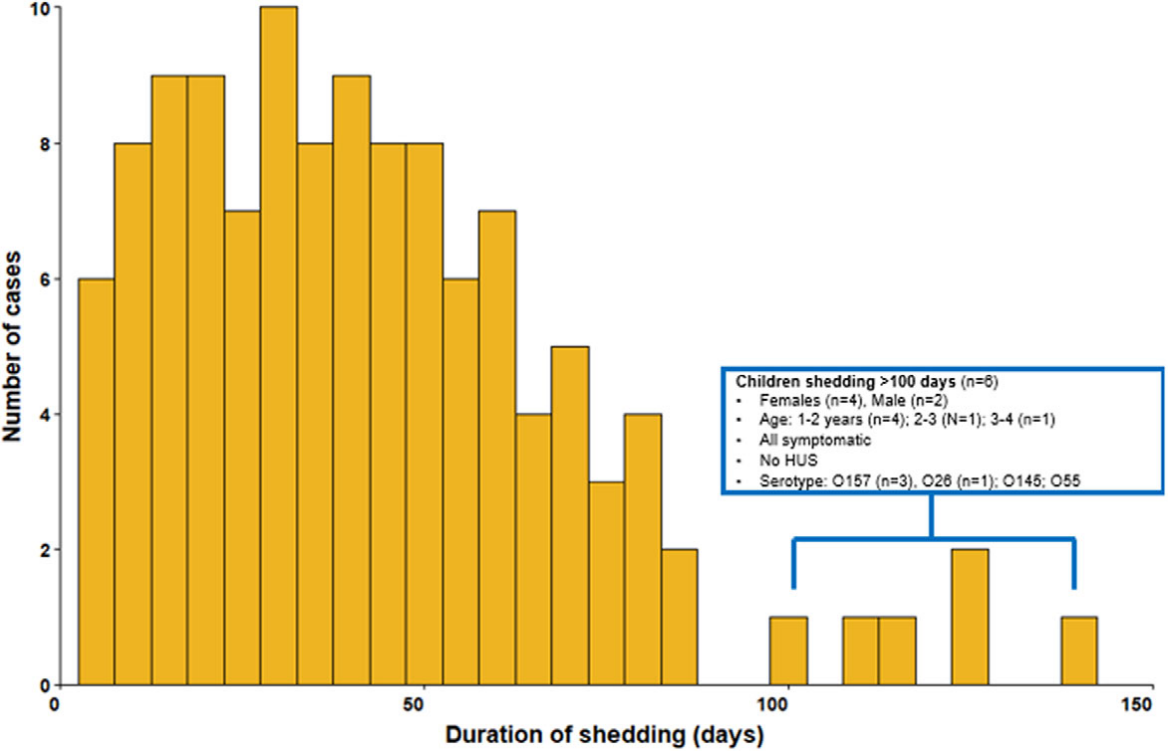
Duration of shedding Shiga toxin-producing *Escherichia coli* in days, England, 2018–2022 among children in childcare settings (*n* = 315).

Duration of shedding could be calculated for 315/367 (86%) cases, and the median was 32 days (IQR 20–44) (Figure 1). Of these, 148 (47%) were shedding for up to 30 days, 137 (43%) for between 31 and 60 days, 24 (8%) continued to shed for 60–100 days, and 6 (2%) for more than 100 days. All six cases who were shedding for >100 days (STEC O157 *n* = 3) were symptomatic but did not develop HUS. Four of these six cases were female, of which three were aged 1–2 years old. Sensitivity analysis excluding those with only sample or report dates of second negative (*n* = 311) produced the same results.

**Figure 2.**
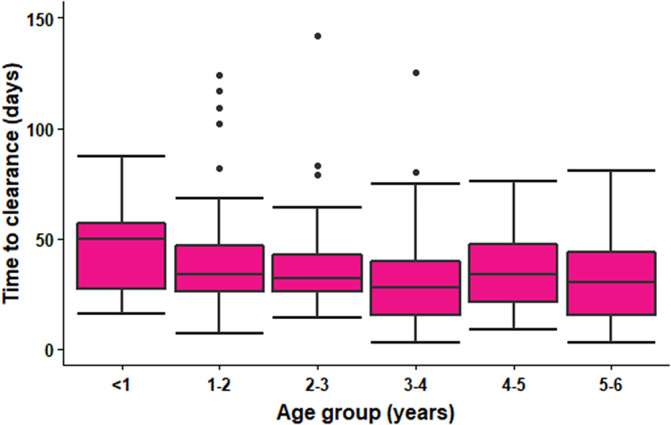
Duration of shedding of Shiga toxin-producing *Escherichia coli* in days by age group of child, England, 2018–2022 (*n* = 315).

**Figure 3.**
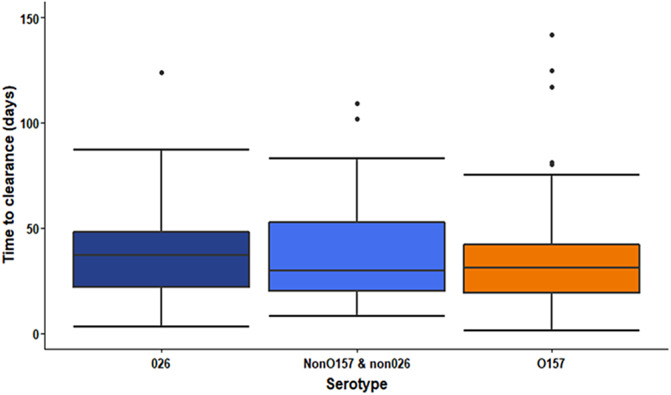
Duration of shedding of Shiga toxin-producing *Escherichia coli* in days by serotype, England, 2018–2022 (*n* = 315).

Younger children shed for longer; duration of shedding decreased by 9% (95% CI 4–13) for every 1 year of increase in age. Data showed boys aged 0–11 months shed for a longer period compared to other age groups (Table 1). There was no significant difference in duration of shedding by serotype (O157 (reference) vs. O26 (1.15, 95% CI 0.95–1.41) vs. non-O26 and non-O157 (1.17, 95% CI 0.89–1.55); Figure 3). Duration of shedding was reduced by 17% (95% CI 4–29) among cases reporting bloody diarrhoea after adjusting for age.

**Table 1.** Duration of shedding of Shiga toxin-producing *Escherichia coli* in days by age group and sex of child, England, 2018–2022 (*n* = 315)

	Female		Male	Total	
Age group (months)	*N*	Median duration of shedding, days (IQR)	*N*	Median duration of shedding, days (IQR)	Median duration of shedding, days (IQR)
0–11	13	30 (26–50)	10	54 (43–62)	50 (26–57)
12–23	26	30 (24–40)	25	36 (28–47)	33 (24–47)
24–35	27	32 (23–44)	31	34 (26–42)	32 (26–42)
36–47	27	28 (15–37)	36	25 (16–38.5)	26 (15–38)
48–59	31	33 (24–55)	36	31 (20–41)	31 (21–46)
60–72	31	28 (14–42)	22	32 (12–44)	28 (13–42)
Total	155	30 (20–44)	160	34 (19.5–45.5)	32 (20–44)

**Figure 4.**
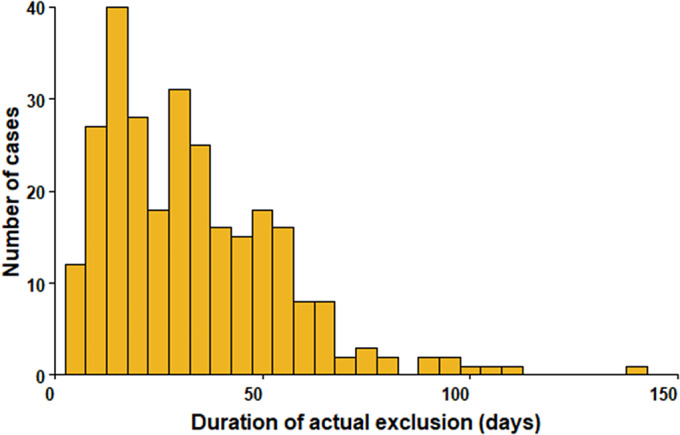
Number of cases by duration of actual exclusion in days, England, 2018–2022 (*n* = 277).

There was no significant difference in duration of shedding by sex, presence or absence of *eae* or *stx* profile of the pathogen, or between cases that had or had not been treated with antibiotics (*n* = 22) or anti-diarrhoeals (*n* = 14). Asymptomatic children cleared infection in a shorter time after the first positive sample (median 18, IQR 8–30) than symptomatic children (median 32, IQR 21–47).

### Exclusion

Nearly all cases (96.7%, 352/364 where information known) were excluded from childcare settings and their median duration of exclusion was 29 days (IQR 16–46, *n* = 277). Sensitivity analysis including only those with a known date of exclusion to calculate median duration of actual exclusion (*n* = 252) produced similar results – median 28 days (IQR 15–46).

In general, actual exclusion periods were shorter than required (based on symptom onset and microbiological clearance). Among cases with available information (*n* = 244), the median duration of actual exclusion (31 days, IQR 18.5–48) was nearly 10 days shorter than the median required exclusion (40.5, IQR 28.5–56). In some cases, implementation of exclusion was delayed; the median duration between symptom onset date and date of exclusion was 10 days (IQR 5–20) in the 195 symptomatic cases who had information available.

The period of actual exclusion and duration of shedding was available in 261 cases. Thirty-four of these cases (13%) were excluded for at least 2 weeks longer than their duration of shedding, the majority being in STEC O157 cases (68%). This was due to a delay in a second sample being taken following an initial negative; the median interval was 6 days (IQR 2–14) in 27 cases with information. Additionally, we found delays in the result being reported to HPTs. Of the 17 cases with sample collection dates and result reported dates; the median interval for these cases was 8 days (IQR 5–9). In general, once the health protection team had the negative result, families were informed promptly that their children could return to childcare. Overall, 80% (129/161) of cases were informed they can return to childcare on the same day as report date of second negative sample.

The most common difficulties in implementing inclusion reported were parental dissatisfaction, financial losses, and working parents (Table 2). Effective communication with parents emerged as the most frequently reported strategy for managing these challenges. Analysis of all free text responses is planned to be reported elsewhere.

**Table 2.** Difficulties in implementing exclusion and ways of managing exclusion

Implementing exclusion*	*N* (%)
*Difficulties* (*n* = 100)	
Parental dissatisfaction	53 (53)
Working parents	33 (33)
Financial losses	30 (30)
Childcare problems	12 (12)
Concerns regarding social isolation	9 (9)
Late exclusion/result	7 (7)
Other	26 (26)
*Managing difficulties with exclusion* (*n* = 81)	
Communication with parents	72 (89)
Increasing frequency of testing	21 (26)
Offers of financial support	4 (5)
Alternative childcare arrangements	4 (5)
Other	15 (19)

* Multiple difficulties and strategies could be reported per case.

### Return to setting prior to clearance

Of excluded cases with available information (288/336), 23% (67/288) returned to the childcare setting prior to clearance. The most common reasons for returning prior to clearance were reassessment of risk (*n* = 27), late exclusion (*n* = 9), family unknowingly sending child back *(n* = 7), and family deliberately sending child back (*n* = 4), in the 65 cases where a reason was documented. The reassessment of risk primarily stemmed from the reference laboratory’s results (provides additional information on *stx* subtypes and serotype), which revealed that the infection was attributed to a lower risk non-O157 STEC strain. Consequently, this indicated that there was no longer a need for case exclusion and microbiological clearance. Other contributing factors included children transitioning to the age of 6 during the exclusion period, no longer falling into a high-risk category, and the implementation of enhanced hygiene and handwashing measures in settings of asymptomatic cases exhibiting prolonged shedding.

### Infectious case at childcare setting

More than half of cases (56% (176/313 where information available)) attended their childcare setting while infectious (symptomatic or while STEC culture or PCR positive). The odds of being infectious at a childcare setting were 3 times higher among 1- to 2-year-olds (OR 3.0, 95% CI 1.4–6.8) compared to over those aged 5 years or older (Table 3).

**Table 3.** ORs of age groups (years) associated with being infectious at a childcare setting

Variable	*N*	OR (95% CI)	*P*-value
*Age group (years)*			
<1	22	1.37 (0.49–3.82)	0.55
1–2	62	3.03 (1.35–6.79)	0.01
2–3	62	1.58 (0.73–3.43)	0.25
3–4	58	1.41 (0.64–3.07)	0.39
4–5	64	1.01 (0.47–2.17)	0.98
5–6	45	Ref	0.00

The number of days the case attended their childcare facility while infectious was recorded by the data collector for 103/176 cases. Of these cases, the median number of days attended was 2 (IQR 1–5), with 5/103 cases attending for more than 2 weeks while infectious, which could be prior or after exclusion by HPT. These five cases attended while infectious due to the following reasons: long-term history of diarrhoea (*n* = 1), reference laboratory results confirming high risk strain received nearly 1 month after onset date (*n* = 2), notification of result to HPT 2 weeks after sample date (*n* = 1), and miscommunication between carers and HPTs and Environmental Health Departments (EHDs) (*n* = 1).

### Risk of secondary transmission to children from an infectious primary case in childcare settings

From the 338 childcare settings in the dataset, we had information about whether cases attended while infectious for 290 (86%). Of those, 168 (57.9%) settings had been attended by a case while infectious, and 9 of those (9/168 (5.4%)) had two or more laboratory confirmed cases. However, 5/9 of these incidents only involved siblings receiving childcare at the same setting and were likely household transmission incidents. Four settings (2.4%) had likely transmission within the setting.

Screening was carried out at 16/168 (10%) settings (close contacts of case *n* = 5; symptomatic children only *n* = 8; everyone *n* = 3). The basis for screening in these settings, when information was available, involved the presence of symptomatic children or staff in the setting or inadequate hygiene standards. Close contacts were identified based on being in the same room at the setting as the case or receiving care from the same staff member as the case. About 4/16 (25%) settings that undertook screening had identified two or more laboratory-confirmed cases.

The linked cases at the four settings involved 18 cases: 4 primary, 2 co-primary, and 12 secondary cases (Table 4). The ratio of secondary to primary/co-primary cases was estimated as 2 (12/6). Of the 12 secondary cases, 7 were asymptomatic. All secondary cases attended the same group or class as the primary/co-primary case. Based on the four incidents where secondary transmission was identified, the secondary transmission rate (STR) among close contacts was 13% (12/92); the STR was nearly 10 times higher among close contacts in nurseries compared to primary school settings.

**Table 4.** Summary of incidents with evidence of transmission

Type of setting	No. of primary cases shedding at facility	No. of days at facility while shedding	No. of confirmed secondary child cases	No. of close child contacts	No. of child contacts (setting)	Screening	Close Contact Secondary transmission rate
Nursery	1 primary; 2 co-primary	Primary: 13 days Co-primary: 15 days + 7 days	4 (S), 3 (A)	22	157	Y (130/157 submitted a sample)	32%
Nursery	1	1	3 (A)	12	100	Y (1st round of screening baby room only – 29 children and 7 staff; 2nd round entire nursery)	25%
Primary school	1	1	1 (S)	30	60 (reception and year 1)	Y (all reception and year 1 as shared toilets)	3.3%
Primary school	1	3	1 (A)	28	No info	Y (3 staff and 2 children close contacts)	3.5%

*Abbreviations: A, asymptomatic; S, symptomatic; Y, yes.*

## Discussion

The study shows that children aged <6 years, attending childcare settings, take approximately 32 days to achieve microbiological clearance of STEC. Additionally, there is no statistical evidence indicating that serotype designation impacts shedding duration.

### Duration of shedding

The shedding duration estimates identified in this study were consistent with previous findings in England (median 31 days, IQR 17–41) [8]. Similar to the 2010–2011 data, we found 24% of children take more than 6 weeks to achieve clearance, with a maximum time period to clearance of 142 days (vs. 135 days in Dabke study) [8].

Our data are consistent with the international evidence that STEC can take a prolonged time to clear [15]. Periods of shedding STEC in children reported outside of the UK range from 10 to 283 days, with lower estimates (median 10–20 days) usually reported by smaller studies (<45 cases) and in outbreak contexts [16–18]. Our estimation of median shedding duration falls within the range of other reported estimates from comparable studies carried out in Sweden (median 20 days, *n* = 165) [13] and Ireland [14]. The Ireland study was a 10-year analysis of children aged <6 years (*n* = 188) and identified a median time of 39 days (IQR 27–56) with a maximum time period of 283 days. The longer median duration reported in the study is likely due to the definition of microbiological clearance as the time between symptom onset (or date of first positive) and the date of the second negative sample, whereas our study used the date of the first negative sample. Similar to our findings, they found no significant impact on clearance by serotype. Additionally, other studies have demonstrated no association between the presence of specific genes tested and the duration of shedding, further supporting our observations [12–14].

### Factors associated with duration of shedding

Consistent with other studies, we found younger children may take more time to achieve microbiological clearance [8, 14] which may be a result of immature immune systems and ongoing development of intestinal flora. Additionally, we found boys aged 0–11 months exhibited a prolonged shedding period in comparison with other age groups; however, sex-based difference in shedding patterns have not been noted previously. Interestingly, we also found those who reported blood in stool achieved clearance quicker than those who did not. We speculate this could be possibly explained by a more aggressive immune response in those with severe symptoms leading to faster and more effective clearance. Similar to Collins et al., we also found asymptomatic children cleared infection in a shorter time than symptomatic children [14]. However, it is not possible to determine the point at which asymptomatic cases were infected and their duration of shedding prior to screening; therefore, this finding is likely artefactual and an underestimate of actual carriage length.

### Exclusion

Our study highlights a median delay of 10 days from symptom onset to formal exclusion by public health staff. Potential explanations for this could be delays in seeking healthcare and therefore sample submission or delays in notification to HPT from laboratories. However, our study shows the limited duration which an infectious case is present within a setting which indicates cases are likely being excluded promptly by the setting/carers after symptom onset.

Although we would expect actual exclusion to be longer than shedding, we found just over 10% of cases were excluded for 2 weeks longer than shedding. Our study showed that communication between HPTs and families was prompt, with most families informed their child could return to their childcare setting within 1 day of the report date of the second negative sample, and unlikely to contribute to the extended exclusion periods. Instead, the data suggest the delay may be due to factors like parents’ delayed submission of second samples (median time between first and second negative sample: 6 days) and possibly delays in transport or laboratory reporting (median time: 8 days between the second negative sample date and reporting of results).

Reassessment of risk was most frequently reported as the reason for returning to the childcare setting before clearance. As highlighted previously [8], parental dissatisfaction with exclusion is a key issue and effective communication with parents is necessary to inform them about the expected duration of shedding and the importance of exclusion in terms of preventing onward transmission.

### Transmission

Our results indicate over half of childcare settings were attended by a case while infectious. However, the overall transmission risk identified was low, at 2.4%. The observed low proportion of transmission events may have been due in part to the current exclusion policy. However, it is likely also due to the varying criteria for initiating screening used by different public health teams within a childcare setting. Only 10% of settings that were attended by a case while infectious, underwent screening and in nearly a quarter of these settings, evidence of transmission was identified. Notably, we observed the close contact secondary transmission rate was nearly ten times higher in nurseries compared to primary school settings, which is in line with previous research and indicative of ease of spread between nursery aged children [19]. Furthermore, in our study, 58% of the secondary cases that were identified were asymptomatic. Although it is not possible to determine the point at which they were infected or their role in transmission as they may have been the index cases, they do provide evidence of transmission within the setting.

To the best of our knowledge, this is the largest study assessing duration of shedding in children attending childcare settings. However, the following limitations must be considered. Children with shorter duration of symptoms and milder symptoms are less likely to be tested and therefore less likely to be included in our study population, which could impact our shedding duration estimates. This study is thus not an accurate description of the biology of clearance but does allow us to predict likely duration following diagnosis in practice, which is important for parents and carers and is comparable to other studies. Furthermore, the estimates reported depend on frequency of testing which we were unable to capture, as cases may in fact be free of carriage between their last positive and first negative sample.

Information quality recorded varied; test method and type of testing laboratory were often missing which may have an effect on exclusion estimates which was based on report date of samples. Probiotic usage was also poorly recorded and necessitates further investigation of their potential impact on shedding duration.

It is very hard to draw conclusions regarding transmission risk within childcare settings when screening is not universal. Screening strategies vary between HPTs and there were few incidents with active screening. Justification as to why active screening was or was not conducted was not always clearly documented. The lack of consistency is likely due to the absence of specific standards and/or criteria for initiating screening. Active screening of contacts is not routinely performed in settings following a single case, since transmission may have also taken place in other settings where children interact. Furthermore, asymptomatic cases may be misclassified as secondary, whereas they could be primary cases. We were also unable to capture setting level risk factors that may influence transmission risk, e.g., hygiene practices, staff training and knowledge, effective policies for excluding children with diarrhoea, food safety, and feeding practices [20].These limitations can affect the accuracy of the transmission risk estimates in this study in opposite directions.

## Conclusion

This is the largest study assessing duration of shedding in children attending childcare settings reported in the literature and based on current diagnostic practices and epidemiology. Our findings suggest that current guidance regarding exclusion and supervised return of prolonged shedders in England remains valid despite recent changes to STEC epidemiology.

Our study has highlighted a number of areas where further investigation is recommended, but this is dependent on systematically collected data being available. Factors such as probiotic usage on duration of shedding need to be further explored to better inform implementation of control measures. Further understanding of potential delays in turnaround times for negative results, alongside effective communication between laboratories and HPTs and EHDs, is required to reduce the exclusion period and ensure the child can resume their education and interaction with peers. Further work to understand the impact of exclusion on children and parents is currently being undertaken and would further help public health teams manage and coordinate the exclusion process with parents.

We also recommend a systematic approach to screening in childcare settings, such as a framework with a checklist to capture rationale for decisions and childcare setting level risk factors for transmission such as hygiene and feeding practices; variability in HPT approach could result in clinical risk with potential for serious harm.

## Supporting information

Vusirikala et al. supplementary material Vusirikala et al. supplementary material

## Data Availability

Data are incorporated into the article and material contained within. Individual-level data cannot be shared due to ethical/privacy reasons. Custom code using Stata17.

## Supplementary material

For supplementary material accompanying this paper visit http://doi.org/10.1017/S095026882400027X. click here to view supplementary material
