# Supplementary material for: Shedding and exclusion from childcare in children with Shiga toxin-producing Escherichia coli, 2018–2022
Source: Epidemiol Infect. 2024 Feb 26;152:e42. doi: 10.1017/S095026882400027X (PMC10945940; doi:10.1017/S095026882400027X)
Supplement: Vusirikala et al. supplementary material [file S095026882400027Xsup001.docx]

**SUPPLEMENTARY MATERIAL**

**Supplementary Figure 1. Flow diagram of study population**


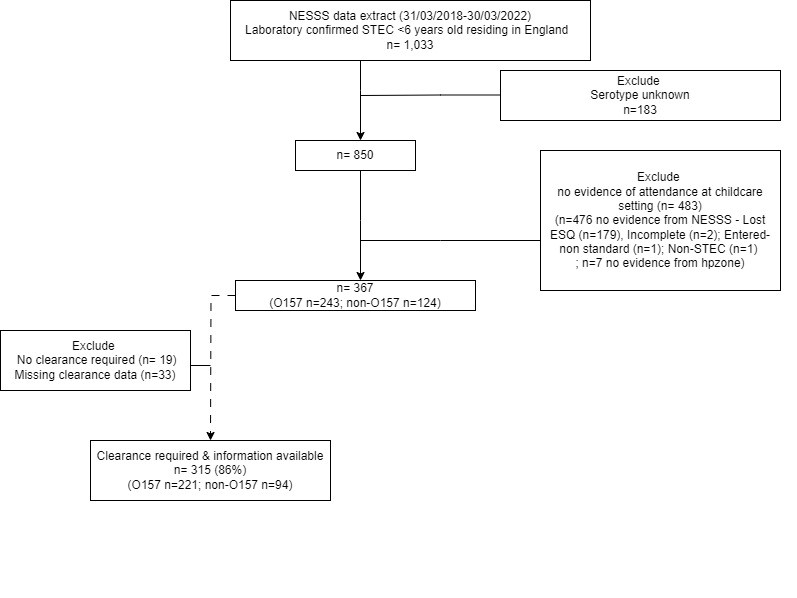


Subset of study population
